# Hydraulic Traits Emerge as Relevant Determinants of Growth Patterns in Wild Olive Genotypes Under Water Stress

**DOI:** 10.3389/fpls.2019.00291

**Published:** 2019-03-13

**Authors:** Virginia Hernandez-Santana, Pablo Diaz-Rueda, Antonio Diaz-Espejo, María D. Raya-Sereno, Saray Gutiérrez-Gordillo, Antonio Montero, Alfonso Perez-Martin, Jose M. Colmenero-Flores, Celia M. Rodriguez-Dominguez

**Affiliations:** ^1^Irrigation and Crop Ecophysiology Group, Instituto de Recursos Naturales y Agrobiología de Sevilla, Consejo Superior de Investigaciones Científicas, Seville, Spain; ^2^School of Agricultural Engineering, CEIGRAM, Universidad Politécnica de Madrid, Madrid, Spain; ^3^Centro “Las Torres-Tomejil”, Instituto Andaluz de Investigación y Formación Agraria y Pesquera, Seville, Spain; ^4^School of Biological Sciences, University of Tasmania, Hobart, TAS, Australia

**Keywords:** hydraulic allometry, leaf hydraulic conductance, leaf:sapwood area ratio, leaf:root area ratio, net photosynthesis rate, stomatal conductance

## Abstract

The hydraulic traits of plants, or the efficiency of water transport throughout the plant hydraulic system, could help to anticipate the impact of climate change and improve crop productivity. However, the mechanisms explaining the role of hydraulic traits on plant photosynthesis and thus, plant growth and yield, are just beginning to emerge. We conducted an experiment to identify differences in growth patterns at leaf, root and whole plant level among four wild olive genotypes and to determine whether hydraulic traits may help to explain such differences through their effect on photosynthesis. We estimated the relative growth rate (RGR), and its components, leaf gas exchange and hydraulic traits both at the leaf and whole-plant level in the olive genotypes over a full year. Photosynthetic capacity parameters were also measured. We observed different responses to water stress in the RGRs of the genotypes studied being best explained by changes in the net CO_2_ assimilation rate (NAR). Further, net photosynthesis, closely related to NAR, was mainly determined by hydraulic traits, both at leaf and whole-plant levels. This was mediated through the effects of hydraulic traits on stomatal conductance. We observed a decrease in leaf area: sapwood area and leaf area: root area ratios in water-stressed plants, which was more evident in the olive genotype *Olea europaea* subsp. *guanchica* (GUA8), whose RGR was less affected by water deficit than the other olive genotypes. In addition, at the leaf level, GUA8 water-stressed plants presented a better photosynthetic capacity due to a higher mesophyll conductance to CO_2_ and a higher foliar N. We conclude that hydraulic allometry adjustments of whole plant and leaf physiological response were well coordinated, buffering the water stress experienced by GUA8 plants. In turn, this explained their higher relative growth rates compared to the rest of the genotypes under water-stress conditions.

## Introduction

One of the main challenges facing the world today is to achieve food security, a problem that is aggravated by climate change, natural resource depletion and adverse impacts of environmental degradation (desertification, drought, freshwater scarcity, etc.) (United Nations, 2015). Promising approaches for ensuring the stability of food production under limited water availability involve breeding practices that take advantage of the genetic variability of wild related species and different cultivars that have better adapted to environmental constraints (Nevo et al., 2012; Burnett et al., 2016), such as water-deficit conditions (Ruane et al., 2008; Reddy et al., 2017; Trentacoste et al., 2018). Crop breeders seek to identify and select traits or mechanisms that enable high biological or reproductive yields to be achieved under water-limited conditions (Turner, 2017). As demonstrated in recent studies, some morphological leaf traits (López-Sampson et al., 2017) or processes such as osmotic adjustment (Blum, 2017) explain a large proportion of a tree species’ growth, which indicates the potential value of focusing on certain traits to help in the selection of the most productive species or varieties.

Specific knowledge of how hydraulic traits of plants (i.e., the efficiency of water transport throughout the plant) limit plant performance could help to anticipate the impact of climate change (Anderegg et al., 2016) and to improve the security and sustainability of our food supply. Nevertheless, the mechanisms explaining the role of hydraulic traits on growth are complex and only just beginning to be elucidated (Sack et al., 2016). Stomatal control of transpiration is directly or indirectly regulated by changes in plant water status, produced by changes in the soil-to-leaf water transport properties (Buckley, 2005). Under water deficit conditions, stomata close to avoid leaf desiccation but in doing so, carbon dioxide uptake is restricted, and in turn, assimilation rate. Thus, growth can be limited by both carbon supply and turgor pressure. Above-ground hydraulic resistances to water flow mainly lie in leaves (Nardini and Salleo, 2000; Nardini et al., 2001, Brodribb and Holbrook, 2003; Sack et al., 2003), creating a positive link between leaf hydraulics and leaf gas exchange (Brodribb and Holbrook, 2004, 2006; Brodribb et al., 2005, 2007; Brodribb and Jordan, 2008; Scoffoni et al., 2016; Reddy et al., 2017; Xiong et al., 2018). In that sense, leaf hydraulics have been suggested to be important to both water and carbon (C) fluxes (Reich, 2014).

These studies highlight the coordination of maximum values of leaf gas exchange and leaf hydraulic conductance, i.e., under steady-state, non-stress conditions. The potential relevance of this coordination to plant performance under water-deficit conditions has also been investigated (Brodribb and Holbrook, 2004; Lo Gullo et al., 2005; Gortan et al., 2009; Chen et al., 2010; Hernandez-Santana et al., 2016). Besides these short-term mechanisms of stomatal control through leaf hydraulics, plants also respond to water stress through processes influencing equilibria and steady-state behaviors across the entire plant system, adjusting their root/shoot functional balance accordingly (Mencuccini, 2014), i.e., changing the hydraulic allometry of the plant. Nevertheless, in response to water stress, more research is needed to quantify responses in relation to plant anatomy, allocation, architecture and physiology (Addington et al., 2006; Martínez-Vilalta et al., 2009; Zhou et al., 2016; Martin-StPaul et al., 2017) to better understand how development is coordinated in different environments based on the underlying mechanisms (Sterck and Zweifel, 2016).

In relation to olive genotypes, very little is known about how hydraulic traits and photosynthetic assimilation rates in response to water stress influence growth. We know that olive species rely on a range of physiological traits and mechanisms to cope with water deficit (Fernández, 2014; Diaz-Espejo et al., 2018). However, to progress breeding efforts, knowledge of genotypic variation for water-use traits and how they influence plant performance under water stress is required. As such, we conducted an experiment that employed both well-irrigated and water-stress conditions to identify differences in growth patterns among different wild olive genotypes, and to determine whether hydraulic traits may help to explain such differences through their effect on stomatal conductance and photosynthesis rate.

Our objectives were: (i) to evaluate whether different relative growth rate (RGR) patterns representing different physiological strategies arise in wild olive genotypes at leaf, root and plant level and to determine the effects of water availability on these growth patterns and (ii) to determine the role of hydraulic traits (mediated by their effect on leaf gas exchange) at the leaf and whole-plant levels to explain differences in RGR patterns at plant scale in two contrasting olive genotypes.

## Materials and Methods

### Experimental Overview

We conducted our experiment using four genotypes (AMK6, ACZ9, GUA6, and GUA8) selected from a first screening of 39 wild genotypes representing three different subspecies of *Olea europaea* (*europaea* var. *sylvestris, guanchica* and *cuspidata*). We assessed the effect of long-term deficit irrigation on growth patterns of these four genotypes, and afterward, we focused on two of them that presented the most contrasting trends in growth (GUA6 and GUA8) to explore the physiological and morphological traits that explained these differences in growth performance. The specific measurements performed during each period of the experiment are provided in Table 1.

**Table 1:** Period, frequency, and number of replicates per genotype and irrigation treatment for the variables measured along the experiment and the genotypes where they were measured.

Measurement period	Dates	Genotypes	Variables	Measurement frequency	Replicates
Harvest 1–Harvest 2	From 06-04-2016 to 05-04-2017	ACZ9	Relative growth rate (RGR, g g^-1^ day^-1^)	Once	Four
		AMK6	Leaf mass fraction (LMF, g g^-1^)	Once	Four
		GUA6	Root mass fraction (RMF, g g^-1^)	Once	Four
		GUA8	Specific leaf area (SLA, m^2^ g^-1^)	Once	Four
			Specific root length (SRL, m g^-1^)	Once	Four
			Net assimilation rate (NAR, g m^-2^ day^-1^)	Once	Four
			Maximum stomatal conductance (*g*_s,max_, mol m^-2^ s^-1^)	Fortnightly-Monthly	Two (2016) and three (2017)
			Maximum net photosynthesis rate (*A*_N,max_, μmol m^-2^ s^-1^)	Fortnightly-Monthly	Two (2016) and three (2017)

After Harvest 2	From 06-04-2017 to 29-08-2017	GUA6	Maximum stomatal conductance (*g*_s,max_, mol m^-2^ s^-1^)	Twice	Four
		GUA8	Maximum net photosynthesis rate (*A*_N,max_, μmol m^-2^ s^-1^)	Twice	Four
			Maximum velocity of carboxylation (V _cmax_, μmol m^-2^ s^-1^)	Once	Four
			Mesophyll conductance (*g*_m_, mol m^-2^ s^-1^)	Once	Four
			Leaf water potential (Ψ_leaf_, MPa)	Twice
			Foliar N (*g*N m^-2^)	Once	Four
			Osmotic pressure at full turgor (Π_0_, MPa) Turgor loss point (TLP, MPa)	Once	Four
			Vulnerability curve of leaf hydraulic conductance (*K*_leaf_, mmol m^-2^ s^-1^ MPa^-1^)	Once	-
			Leaf:sapwood area (cm^2^ mm^-2^)	Once	Four
			Leaf:root area (m^2^ m^-2^)	Once	Four

#### Screening of 39 Wild Olive Genotypes Before Harvest 1

The seeds for this first screening were obtained from trees located in the World Olive Germplasm Collection of Córdoba (Spain) and Grahamstown (South Africa). The plants were propagated and rooted *in vitro* from zygotic embryos obtained from the prospected seeds during 2014. Seeds were obtained by breaking olive pits with a tube cutter and surface sterilized with hypochlorite. Sterile embryos were obtained from the seeds and placed in test tubes with hormone-free olive medium (Rugini, 1984). After *in vitro* germination, the genotypes were multiplied through propagation of nodal segments in Rugini medium supplemented with 1 mg L^-1^ zeatin (Rugini, 1984). The explants were kept in an *in vitro* culture chamber at 25°C and subjected to a photoperiod of 16 h light/8 h darkness, using LED illumination 70% red plus 30% blue (70/30) with 34 μmol m^-2^ s^-1^ of photosynthetic photon flux. Plants were rooted in 1/2x Rugini medium supplemented with 0.8 mg/L Naphthaleneacetic acid for 3 weeks. After *ex vitro* acclimatization, plants were grown under greenhouse conditions for 9 months in 1 L pots. Healthy and homogenous plants were selected and transplanted into 10 L pots containing vermiculite:peat:perlite substrate (40:40:20) and acclimatized for a further 2 months. The 39 genotypes were evaluated during 2015 to assess their water use and fresh weight below and above-ground components as well as the whole plant. For each of these 39 genotypes, six well-irrigated plants (100% field capacity) and six water-stressed plants (60% field capacity) were maintained. Every 2–3 days water loss was quantified and plants were re-watered up to their corresponding water status. Plants were harvested at the end of the trial, and the fresh and dry weights of shoots (leaves and stems) and roots were recorded.

#### Experimental Management During the Measurements Performed in AMK6, ACZ9, GUA6, and GUA8 From Harvest 1 to Harvest 2

**FIGURE 1:**
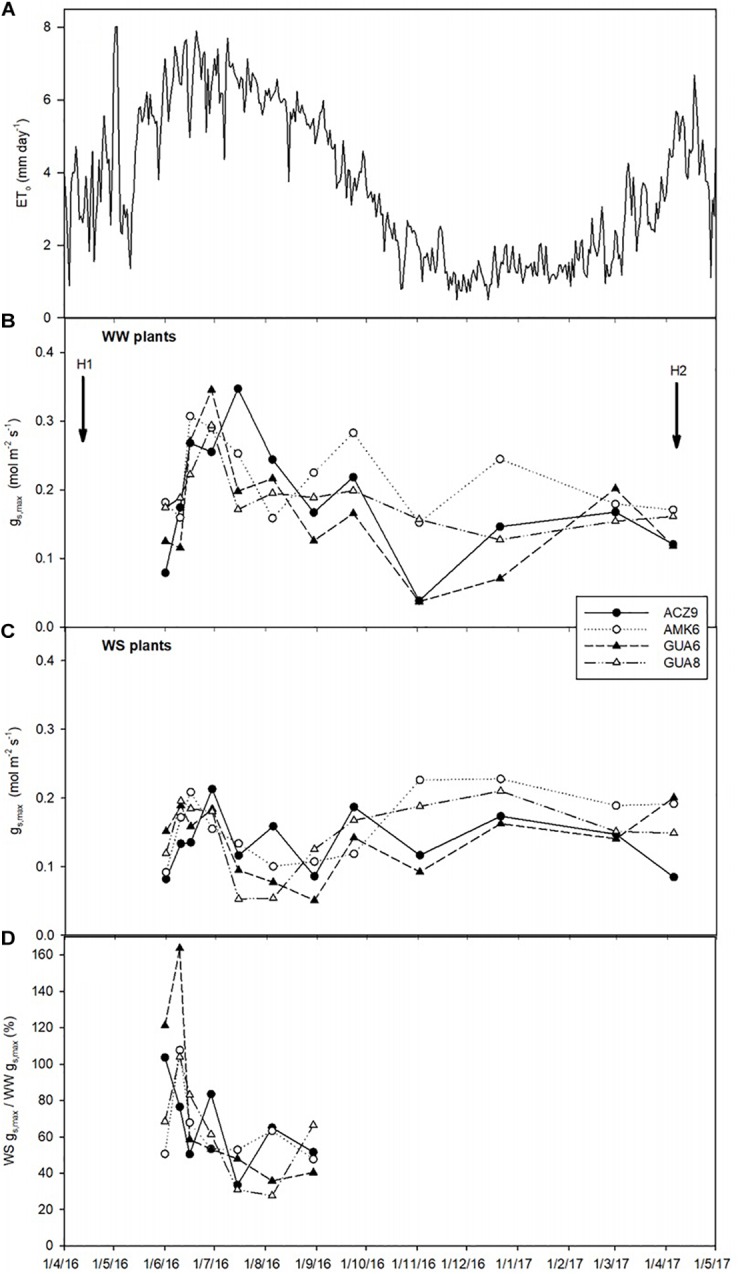
Temporal dynamics of reference evapotranspiration, ET_o_ **(A)**, maximum stomatal conductance (*g*_s,max_) for the different genotypes for well-watered plants (WW) **(B)**, water-stressed plants (WS) **(C)**, and percent of WS compared to WW *g*_s,max_ **(D)** (only June–August data shown for clarity purposes). Each data-point in 2016 represents the average of two plants, while from January to April 2017 the average of three plants is used. H1 and H2 indicate when harvests 1 and 2 took place.

We selected these four genotypes because they presented contrasting behaviors to water deficit in terms of water use and plant, shoot and root fresh weight (Supplementary Figure S1). Plants from these four genotypes were grown outdoors in 25 L pots in La Hampa CSIC experimental orchard, near Seville (Spain) (37°17′N, 6°3′W, altitude 30 m), filled with soil (sandy loam) from this orchard. The size of the pot was not limiting for plant growth. This was based on the observation that roots did not grow enough to fill the entire volume of the pots and some parts of the soil were not explored by them at the end of the experiment. The pots were distributed randomly in rows of 20 plants at 1 × 1.5 m, alternating well-watered (WW) rows and water-stressed rows (WS). This distribution was sufficient to avoid shading by neighboring plants (based on *in situ* observations). Initial sizes of the plants are shown in Table 2 and although sizes were different among the groups, we calculated growth using RGR, which uses initial and final sizes, to minimize size dependent effects (Hunt et al., 2002). The experiments lasted for 19 months (from February 2016 to August 2017) including the measurements specifically performed only in GUA6 and GUA8 (see next section). The plants of all genotypes were the same age (16 months) when the experiment started. Plants were well-irrigated from February 18 to April 26, 2016. After this date they were irrigated differently until the end of the experiment: WW plants, in which plants were irrigated daily to non-limiting soil water conditions to achieve the highest possible stomatal conductance (*g*_s,max_); and WS, in which plants were irrigated to a level representing 40% of the *g*_s,max_ measured in WW plants throughout the experiment to achieve a moderate water-stress status. To achieve these values of *g*_s,max_, we conducted regular gas exchange measurements and modified the irrigation schedule accordingly (Figure 1), i.e., reducing or increasing the frequency and time of irrigation to change the total amount of water depending on WS *g*_s,max_ values compared to WW *g*_s,smax_. Reference evapotranspiration (ET_o_) was collected from a nearby standard weather station (37°13′N, 6°8′W) belonging to the Agroclimatic Information Network of the local government (Junta de Andalucía, Spain). Two harvests were conducted: on April 6, 2016 after a period when all the plants were well irrigated (harvest 1, H1) and on the April 5, 2017, to assess the effect of the long-term deficit irrigation treatment on the olive plants (harvest 2, H2).

**Table 2:** Average and standard error of the leaf area, basal diameter and maximum height of the plants used in the beginning of the experiment (H1).

		Leaf area (cm^2^)	Basal diameter (mm)	Maximum height (cm)
WW	ACZ9	370.45 ± 27.87	6.16 ± 0.35	92.92 ± 2.47
	AMK6	89.81 ± 15.45	4.74 ± 0.39	51.82 ± 7.07
	GUA6	178.49 ± 18.58	4.51 ± 0.29	72.27 ± 9.49
	GUA8	59.09 ± 15.92	3.47 ± 0.14	20.84 ± 6.08
WS	ACZ9	518.15 ± 28.42	6.37 ± 0.23	99.92 ± 10.33
	AMK6	99.59 ± 10.78	4.59 ± 0.22	53.72 ± 4.11
	GUA6	257.13 ± 13.95	5.68 ± 0.20	83.02 ± 5.64
	GUA8	47.75 ± 12.11	2.99 ± 0.23	17.70 ± 3.26


#### Experimental Management During the Measurements Performed in GUA6 and GUA8 After Harvest 2

After H2, 20 pots of GUA6 and GUA8 were kept under the described irrigation treatments (WS and WW) at the same field experimental site prior to conducting leaf hydraulic conductance measurements, pressure–volume curves and photosynthetic response curves, together with additional gas exchange, plant water status and morphological measurements (Table 1).

### Growth Parameters (AMK6, ACZ9, GUA6, GUA8, From Harvest 1 to Harvest 2)

Plant growth was determined by harvesting four plants per genotype and irrigation treatment (*n* = 4) at H1 and H2. Before harvesting, basal stem diameter was measured to estimate sapwood area (m^2^). After harvesting, total plant leaf area (m^2^) was determined using a Li-Cor 3000-A area meter (equipped with a LI-3050C Transparent Belt Conveyor; Li-Cor, Lincoln, NE, United States). To calculate the biomass (g) of roots, stems and leaves, each component was separated and oven-dried at 60°C for at least 2 days. The following plant traits were calculated for each harvest based on the material obtained: leaf mass fraction (LMF, g g^-1^), root mass fraction (RMF, g g^-1^), specific leaf area (SLA, m^2^ g^-1^) and specific root length (SRL, m g^-1^). The data from each genotype and irrigation treatment for the two consecutive harvests were used to compute the net assimilation rate (NAR; g m^-2^ day^-1^) and the RGR (g g^-1^ day^-1^) for the plant (RGR_plant_), roots (RGR_root_) and leaves (RGR_leaf_) according to Hunt et al. (2002):

(1) RGR= NAR × SLA × LMF

Each component was calculated as follows:

(2) (1W)(dWdt) = (1LA)(dWdt) × LALW× LWW

where *t* is time between harvest 1 and 2, *W* is total dry weight per plant, *L*_A_ is total leaf area per plant and *L*_W_ is total leaf dry weight per plant.

### Root Length and Area (AMK6, ACZ9, GUA6, GUA8, From Harvest 1 to Harvest 2)

The root samples were separated into two groups: fine roots or roots thinner than 2 mm and roots thicker than 2 mm. From the first group of roots (thinner than 2 mm), roots were randomly subsampled, scanning 10% of total biomass using the WinRHIZO system (Regent Instruments, Québec, Canada). The roots thicker than 2 mm were not considered in this analysis as fine roots constitute the primary exchange surface between plants and soil (Jackson et al., 1997). The scanning enabled us to directly obtain the root length (cm) and root area (cm^2^) through the WinRHIZO software.

### Field Gas Exchange Measurements (AMK6, ACZ9, GUA6, GUA8, From Harvest 1 to Harvest 2)

To verify our irrigation treatments, maximum stomatal conductance (*g*_s,max_ mol m^-2^ s^-1^) and net photosynthesis rate (*A*_N,max_, μmol m^-2^ s^-1^) were measured at ∼10.30–11.30 GMT from May 2016 to April 2017 (H2) with a portable gas analyzer (Li-6400; Li-Cor, Lincoln, NE, United States) using a 2 × 3 cm standard clear-top chamber under ambient light, vapor pressure deficit and CO_2_ conditions in healthy, sunny leaves. Preliminary measurements demonstrated that *g*_s,max_ occurred at this time of the day. During this period, gas exchange was measured fortnightly during the summer months, and once every month during the rest of the year. In 2016 we measured gas exchange in one leaf from each of two plants of every genotype and for the two irrigation treatments (*n* = 2). From January 2017 to April 2017 we increased the number of sampled plants to three (*n* = 3).

### Field Gas Exchange and Leaf Water Potential Measurements (GUA6, GUA8, After Harvest 2)

In addition to monitoring *g*_s,max_, gas exchange was measured once in June and July 2017 together with leaf water potential measurements to have concurrent measurements of both variables for the GUA6 and GUA8 genotypes (four plants per irrigation treatment and genotype).

Leaf water potential (Ψ_leaf_) was undertaken immediately after gas exchange measurements with a Scholander-type pressure chamber (Soilmoisture Equipment Corp., Santa Barbara, CA, United States) in one fully expanded leaf per plant.

### Leaf Hydraulic Vulnerability Curves (GUA6, GUA8, After Harvest 2)

Leaf hydraulic conductance (*K*_leaf_, mmol m^-2^ s^-1^ MPa^-1^) was measured after H2 (June of 2017) in fully developed, current year and sun-exposed leaves of WW plants of the GUA8 and GUA6 genotypes to obtain leaf hydraulic vulnerability curves (Ψ_leaf_ -*K*_leaf_). To measure *K*_leaf_, we used the Evaporative Flux Method (EFM, Scoffoni et al., 2012), with the results obtained by this method being similar to *K*_leaf_ measurements in olive achieved by the Dynamic Rehydration Method (DRKM, Blackman and Brodribb, 2011) as demonstrated by Hernandez-Santana et al. (2016). Briefly, the method consists of measuring the flow rate of water through the leaf (mmol m^-2^ s^-1^) and the corresponding Ψ_leaf_. To achieve this, we sealed the pots containing the plants at the field in dark plastic bags containing wet paper towels inside to create a low-demand atmosphere. The plants were left to equilibrate at the laboratory for at least 30 min and then, to measure the leaf water flow, leaves were cut from the bagged plants under purified water and rapidly connected to a flowmeter consisting of silicon tubing containing purified, degassed water. The tubing was connected to a pressure transducer (PX26-005GV, Omega Engineering Ltd., Manchester, United Kingdom), which, in turn, was connected to a Campbell data logger CR1000 (Campbell Scientific Ltd., Shepshed, United Kingdom) which recorded water flow readings every 1 s. Reference tubing of different resistances was used to minimize measurement errors (Sack et al., 2011; Melcher et al., 2012). Once connected, the leaves were allowed to transpire inside a Li-Cor 6400-22 Opaque Conifer Chamber for at least 30 min with the photosynthetically active radiation (PAR) level set to 1,200 μmol m^2^ s^-1^ using the Li-Cor 6400-18A RGB Light Source (both instruments were from Li-Cor, Lincoln, NE, United States) until the water flow was stable (coefficient of variation < 5% for the last 5 min). We chose EFM because using the Li-6400 gas analyzer also allowed us to measure the water vapor flux simultaneously with the liquid water flow. When both gas and liquid flows reached a steady state, leaves were removed from the tubing and stored for equilibration in dark and halted transpiration conditions for at least 30 min. Then, Ψ_leaf_ was measured with a Scholander-type pressure chamber (PMS Instrument Company, Albany, OR, United States). The plants were left to gradually dehydrate in the field so that a wide range of hydraulic conductance and Ψ_leaf_ values were obtained.

### Pressure–Volume Curves: Turgor Loss Point and Osmotic Pressure at Full Turgor (GUA6, GUA8, After Harvest 2)

We used one leaf from four plants for each irrigation treatment (WW, WS) and genotype (GUA6 and GUA8) to calculate pressure–volume curves (*n* = 4). Leaves were sampled in the morning of August 29, 2017 and were rehydrated for 24 h, then left to desiccate. Leaf weight and Ψ_leaf_ were measured many times during that desiccation period until the leaves reached minimum Ψ_leaf_ values of ca. -5 MPa. The turgor loss point (TLP, MPa) and osmotic pressure at full turgor (Π_0_, MPa) were calculated according to Sack and Pasquet-Kok (2017).

### Photosynthetic Response Curves (GUA6, GUA8, After Harvest 2)

Four *A*_N_–*C*_i_ response curves (the response of net CO_2_ assimilation to varying intercellular CO_2_ concentration) were measured between 09:00 and 13:00 GMT on different days in July 2017 for the GUA6 and GUA8 genotypes and for each irrigation treatment (WW and WS) (four repetitions, 16 curves per genotype). Measurements were made using two LI-6400 portable photosynthesis systems (LI-COR, Lincoln, NE, United States) at 28°C (close to ambient temperature), saturating photosynthetic photon flux density (1,600 μmol m^-2^ s^-1^) and an ambient CO_2_ concentration (*C*_a_) of between 50 and 1,150 μmol mol^-1^. After steady-state photosynthesis had been achieved (usually after 20–40 min exposure to saturating PPFD), the response of *A*_N_ to varying *C*_i_ was measured by lowering *C*_a_ stepwise from 400 to 50 μmol mol^-1^, returning to 400 μmol mol^-1^ and then increasing *C*_a_ stepwise from 400 to 1,150 μmol mol^-1^. Each *A*–*C*_i_ curve comprised 16 measurements. The maximum carboxylation rate (*V*_cmax_, μmol m^-2^ s^-1^), maximum rate of electron transport (*J*_max_, μmol m^-2^ s^-1^) and mesophyll conductance to CO_2_ (*g*_m_, mol m^-2^ s^-1^) were estimated by the curve-fitting method proposed by Ethier and Livingston (2004). Prior to curve analysis, CO_2_ leaks in the chamber were corrected by following the procedure described in Flexas et al. (2007). Rubisco kinetic parameters were taken from Bernacchi et al. (2002). Values of *V*_cmax_, *J*_max_ and *g*_m_ obtained from the *A–C*_i_ curve analysis were recalculated at 25°C using the temperature dependence parameters specific for olive reported in Diaz-Espejo et al. (2006, 2007).

### Foliar N (GUA6, GUA8, After Harvest 2)

Leaf samples were taken for N analysis from the H2 samples of all the genotypes and irrigation treatments. Enough current-year leaves were sampled to have at least 0.4 g of dry weight to analyze leaf N. Samples were washed in distilled water, dried at 70°C until constant weight, ground and passed through a 500 mm stainless-steel sieve. N concentration was determined by Kjeldahl method.

### Data Processing and Statistical Analysis (AMK6, ACZ9, GUA6, GUA8)

Statistical analyses were performed to assess the effect of the irrigation treatment and genotype on leaf, root and the whole-plant RGR values, in addition to LMF, RMF, SLA, SRL, and NAR for H2. *V*_cmax_, *g*_m_, Π_0_, TLP, foliar N, leaf area – root area ratio (LA:RA) and leaf sapwood area ratio (LA:SA) were also estimated for the GUA6 and GUA8 genotypes after H2. One-way ANOVA was used in cases where more than two levels were compared, while the Student’s *t*-test was used for comparisons between two levels. No transformations were needed to achieve normality. Differences were considered significant for values of *p* < 0.05. SigmaPlot software (version 12.0, Systat Software, Inc., San Jose, CA, United States) was used to conduct these analyses and provide best-fit curves to the dataset to determine the relationships between the different variables analyzed. In addition, two-way ANOVA was used to analyze the interaction between irrigation treatment and genotype RGR at leaf, root and plant level. We used a mixed model in which we included genotype, irrigation treatment and the interaction between both variables. Finally, we also used analysis of covariance (ANCOVA) that included the interaction term of each component related to RGR with irrigation treatment to test its effect on the relationships established for RGR. For these analyses we considered that variables were linearly related. These analyses were conducted with R software (R Core Team, version 3.4.3, 2018) using the “lm()” function.

Path analysis (structural equation modeling with no latent variables) was used to compare four alternative conceptual models to reveal the causal relationships that link hydraulic variables with *A*_N_ through their effect on *g*_s_. We stated *a priori* the relationships among variables with a strong mechanistic or well-established and accepted empirical basis only (Shipley, 2000). The main underlying hypotheses were: (i) *A*_N_ is determined mainly by *g*_s_, *g*_m_, and *V*_cmax_ (Niinemets et al., 2009; Flexas et al., 2014; Perez-Martin et al., 2014); (ii) *g*_s_ is influenced by leaf hydraulic conductance (Brodribb and Holbrook, 2004; Sack and Holbrook, 2006; Scoffoni et al., 2016), LA:SA and LA:RA (Magnani et al., 2002; Addington et al., 2006; Martínez-Vilalta et al., 2009); and (iii) the major determinant of *V*_cmax_ is foliar nitrogen (Walcroft et al., 1997; Diaz-Espejo et al., 2006, 2007; Niinemets, 2012). We compared four models that differed according to whether *K*_leaf_, LA:SA and LA:RA influence g_s_ directly (see Figure 4A), LA:SA and LA:RA are covariates (see Figure 4B), LA:RA effects on *g*_s_ are mediated by LA:SA (see Figure 4C) and LA:RA and LA:SA influence *g*_s_ through their impact on *K*_leaf_ (see Figure 4D). For the path analysis we have a total of 16 data points obtained from 16 plants, for each variable: 4 replicates × 2 genotypes × 2 treatments. All variables were measured or estimated on each of the 16 plants. To perform this analysis it is not important to consider or compare treatments or genotypes, but to provide estimates of the magnitude and significance of hypothesized causal connections between sets of variables. Although our small sample size (16 points for each variable) limits the complexity of the models and the strength of our conclusions, the results on how the hydraulic variables are related to each other and to *g*_s_ complement the simple regression analyses and comparisons conducted. All regression, covariance and variance relationships were determined and are shown in path diagrams. Gas exchange data used for the analysis were those measured in the *A*–*C*_i_ curves: average *g*_s_ and *A*_N_ obtained at 400 ppm CO_2_ and vapor pressure deficit between 1.5 and 2 kPa. Leaf hydraulic conductance was estimated using the vulnerability curves and Ψ_leaf_ measured for those same plants around the time the data for the curves were obtained. The remaining variables were measured or calculated with the data from each plant. All variables were Ln-transformed before analysis to obtain linear relationships because structural equation modeling assumes linearity between variables and (approximate) multivariate normality (Shipley, 2000). Each path model was fitted and compared with the observed results using maximum likelihood. We conducted a Confirmatory Factor Analysis to test whether the Fit Indices of the model were acceptable in terms of similarity between observed and predicted matrix [*P*-value (chi-square) > 0.05], discrepancy adjusted for sample size [Comparative Fit Index (CFI) > 0.9], and residuals of the model [Root Mean Square Error of Approximation (RMSEA < 0.06)]. Path analyses were conducted and diagrams prepared using the R packages “lavaan” (Rosseel, 2012) and “semPlot” (Epskamp, 2013).

## Results

### Variability in Plant Relative Growth Rates Among Genotypes (AMK6, ACZ9, GUA6, GUA8) and Irrigation Treatments

Due to the deficit irrigation, *g*_s,max_ in the WS plants of the four genotypes selected was lower (around 37%) than that in WW plants, but only for the hottest and driest months (mid-June to September of 2016) (Figure 1B–D). In the remaining months, due to the lower evaporative demand of the experimental site (Figure 1A), the reduced irrigation applications were not sufficient to produce a marked reduction of *g*_s,max_.

**FIGURE 2:**
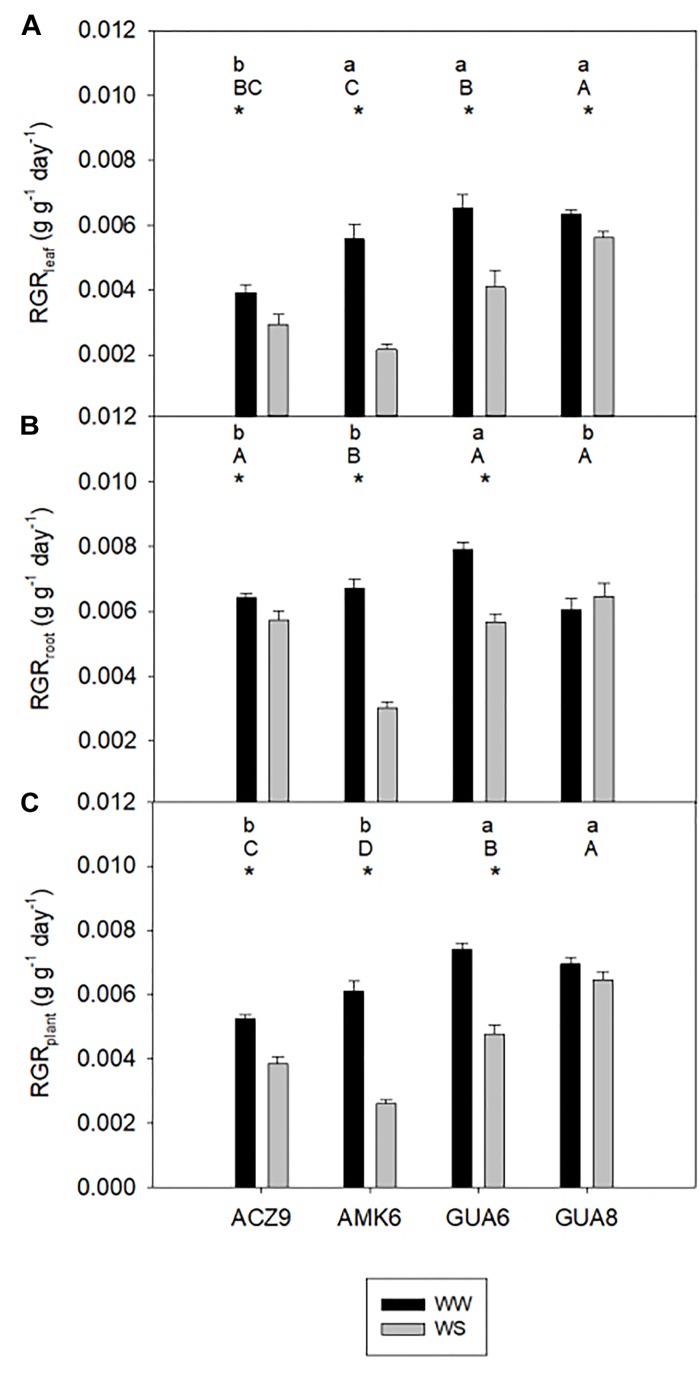
Comparison of relative growth rate (RGR) from harvest 1 to harvest 2 of leaf **(A)**, root **(B)**, and the whole-plant **(C)** biomass for the different genotypes of well-watered plants (WW) and water-stressed plants (WS). Bars are the average of four plants ±1 SE. Lowercase letters indicate statistical differences among the genotypes for WW plants and capital letters for WS plants. The asterisks indicate significant differences (*p* < 0.05) between WW and WS plants for each genotype.

Although the irrigation protocol based on the reduction of *g*_s,max_ provoked only moderate water stress conditions in the hottest months, it was enough to decrease RGR values of WS plants significantly and to different extents amongst genotypes compared to WW plants for the period from April 2016 (H1) to April 2017 (H2) in all genotypes (Figure 2). At the three levels considered, leaf, root and plant, there was a statistically significant interaction between the irrigation treatment and genotype (*p* < 0.001), i.e., the effect of irrigation depends on the genotype. Whereas GUA6 showed the highest RGR in WW plants, both in leaves (6.54 × 10^-3^± 0.42 × 10^-3^ g g^-1^ day^-1^; Figure 2A) and roots (7.90 × 10^-3^± 0.23 × 10^-3^ g g^-1^ day^-1^; Figure 2B), GUA8 presented the highest RGR in WS plants. Moreover the RGR_root_ of GUA8 was statistically similar (*p* > 0.05) between treatments, in contrast to the rest of the genotypes where RGR_root_ was significantly lower in WS than in WW plants (*p* < 0.05). Based on these findings, GUA8 was the genotype in which RGR was least affected by water stress.

**FIGURE 3:**
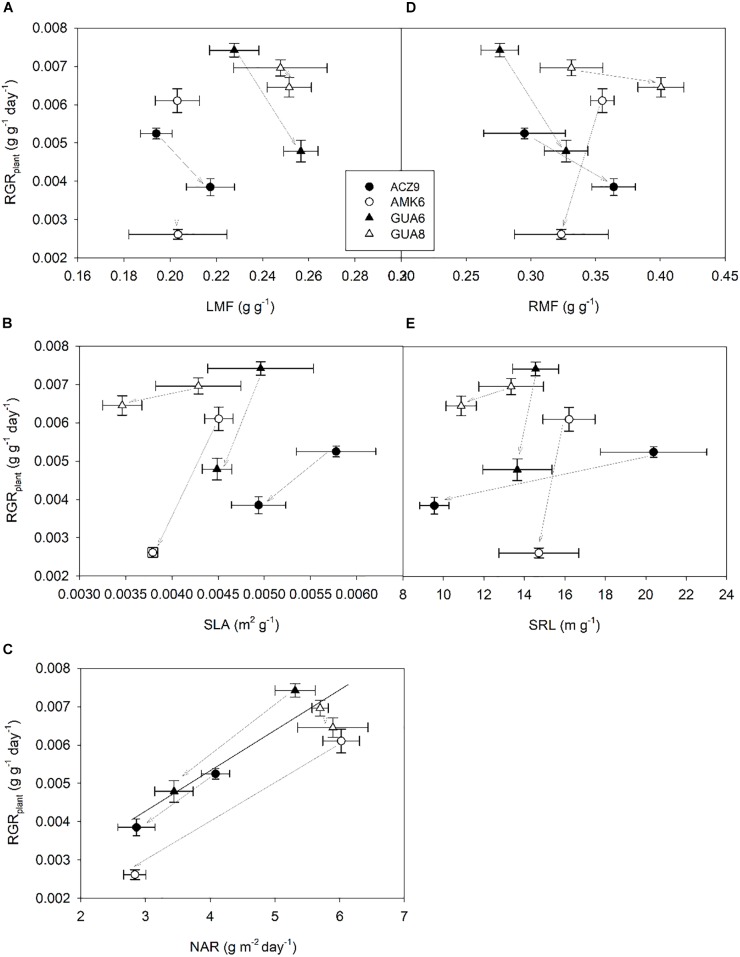
Relationship between plant relative growth rate (RGR_plant_) (H1 to H2) and leaf mass fraction; LMF **(A)**, specific leaf area; SLA **(B)**, net assimilation rate; NAR **(C)**, root mass fraction; RMF **(D)** and specific root length; SRL **(E)** for the different genotypes for well-watered (WW) and water-stressed (WS) plants. Each point is the average of four plants ±1 SE. Gray arrows indicate the change provoked by water stress (from WW to WS plants).

A regression analysis was conducted to relate RGR_plant_ with each of its components at the leaf level (LMF, SLA, and NAR) and corresponding parameters at the root level (RMF and SRL) (Figure 3) by pooling together all genotypes and irrigation treatments. Variations in RGR_plant_ were mainly explained by changes in NAR (Figure 3C) based on the strong correlation between parameters (*R*^2^ = 0.79; *p* < 0.01). The highest RGR_plant_ values were found for those genotypes with the highest NAR. ANCOVA revealed non-significant differences in the regression lines between WW and WS plants. The other traits studied related to carbon allocation (LMF and RMF) and anatomy (SLA and SRL), both for leaves and roots, were not significantly correlated with RGR_plant_. While SLA and SRL showed similar patterns in each genotype, with both parameters reduced under WS conditions (Figure 3B,E), the different magnitude of the change for each genotype prevented a common trend from being identified.

### The Role of Hydraulic Traits in GUA8 and GUA6 to Explain Relative Growth Rate Patterns

**FIGURE 4:**
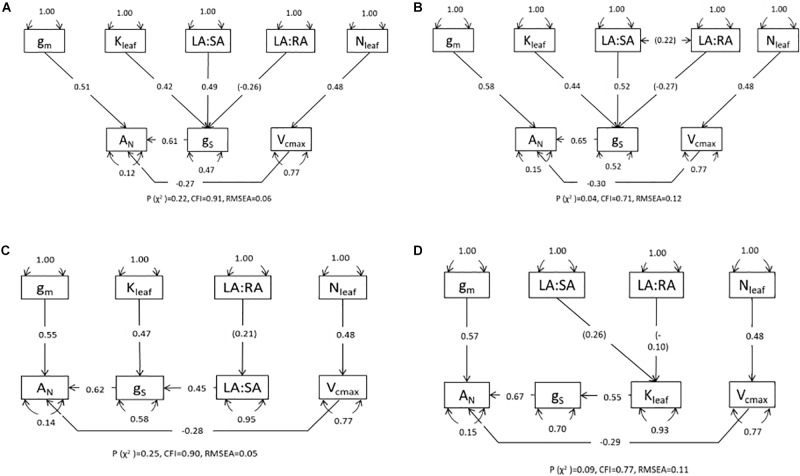
Path diagrams for GUA6 and GUA8 from the plants measured after harvest 2 for the four models describing causal relationships with photosynthesis (*A*_N_) mediated by stomatal conductance (*g*_s_): **(A)** direct impact of leaf hydraulic conductance (*K*_leaf_), leaf area to sapwood area ratio (LA:SA) and leaf area to root area ratio (LA:RA); **(B)** same as in **(A)** but both ratios represented as covariance; **(C)** direct effect of *K*_leaf_ but indirect effect of LA:RA mediated by LA:SA; and **(D)** indirect effect of LA:RA and LA:SA through their effect on *K*_leaf_. Single-ended arrows and associated number indicate direct relationships and standardized parameter estimates of regression; double-ended arrows represent covariance; the curved double-ended arrows are the variances of each variable. In brackets are the non-significant parameters. *V*_cmax_, maximum velocity of carboxylation; *g*_m_, mesophyll conductance; *N*_leaf_, foliar nitrogen. Overall fit statistics for each path model [*p* (χ^2^), CFI (Comparative Fit Index) and RMSEA (Root Mean Square Error of Approximation)] are shown at the bottom of each diagram.

To further explain the above results showing NAR as the main parameter related to changes in RGR_plant_, we focused on gas exchange dynamics at the leaf level of the GUA6 and GUA8 genotypes, including both irrigation treatments, which provided the most contrasting results in terms of growth for the different irrigation treatments. While GUA6 had the highest RGR under the WW conditions (although not significantly so), the same was true for GUA8 under the WS conditions. Pooling together the data for GUA6 and GUA8 to conduct a Path Analysis, we found two path models (Figure 4A,C) that were better than the other two (Figure 4B,D) in terms of fit statistics (see Materials and Methods section for further details). These two best-fitting models differed from each other in terms of how LA:RA impacted on *g*_s_. In the model shown in Figure 4A the effect is direct, whereas in Figure 4C the impact is indirect and mediated by LA:SA. Here, the total variance explained for *A*_N_ was 0.88 and 0.85 for the models in **a** and **c**, respectively. The regression between LA:RA and *g*_s_ or LA:SA was not significant in any case, meaning that LA:SA and *K*_leaf_ were the main variables controlling *g*_s_. The path coefficient was lower in model **a** for *K*_leaf_ (0.42) than for the LA:SA path coefficient (0.49), whereas in model **c** this trend changed slightly (0.47 and 0.45 for the *K*_leaf_ and LA:SA path coefficients, respectively). Stomatal conductance was the variable determining *A*_N_ to the greatest extent across the models.

**FIGURE 5:**
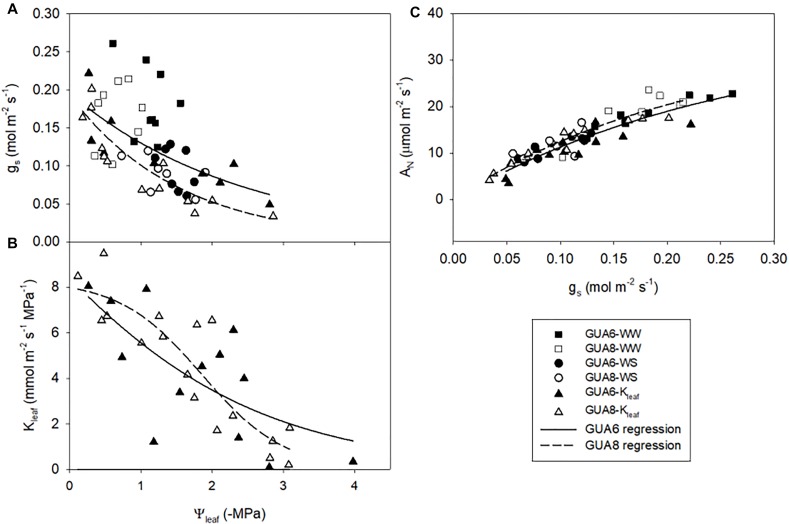
Response curves of stomatal conductance; *g*_s_ **(A)** and leaf hydraulic conductance; *K*_leaf_ **(B)** to leaf water potential Ψ_leaf_; and relationships between *g*_s_ and net photosynthesis rate; *A*_N_ **(C)** for GUA6 and GUA8 genotypes combing information for both well-watered (WW) and water-stress (WS) plants. Each point is one measurement per plant. Data in panels **(A,C)** were obtained from field-conducted *A*–*C*_i_ measurements with corresponding Ψ_leaf_ measurements (June and July 2017); and from the dry-down experiment to obtain the leaf hydraulic vulnerability curves shown in panel **(B)**.

At the leaf level, we further assessed differences in gas exchange and related variables for the different genotypes and irrigation treatments. We observed that *g*_s_ was slightly higher in GUA6 than in GUA8 for all levels of leaf water potential (Figure 5A), and that *A*_N_ was similar between genotypes for all levels of *g*_s_ (Figure 5C). As a result, the water-use efficiency calculated for GUA8 was also higher than for GUA6, in the sense that, to assimilate 1 μmol of CO_2_, GUA6 plants transpired more water than GUA8. Accordingly, *g*_m_ was higher for GUA8 than GUA6 (Table 3), with this difference more evident in WS plants (*p* < 0.05; GUA6: 0.15 ± 0.03 mol m^-2^ s^-1^; GUA8 0.24 ± 0.02 mol m^-2^ s^-1^). In addition, *V*_cmax_ and foliar N (Table 3) followed the same trends for *g*_m_, with statistically significant differences (*p* < 0.05) seen in the foliar N of WS plants. The hydraulic vulnerability curves for both genotypes were similar over the range of Ψ_leaf_ values and followed a sigmoidal shape (Figure 5B). No significant differences between irrigation treatments or genotypes were seen for the other hydraulic traits (Π_0_ and TLP) analyzed (Table 3).

**Table 3:** Average and standard errors of different variables measured in the genotypes GUA6 and GUA8, for well-watered (WW) and water-stressed plants (WS).

	GUA6		GUA8	
		
	WW	WS	WW	WS
Foliar N	3.68 ± 0.46	**3.71 ± 0.46**	3.92 ± 0.99	**5.83 ± 0.78**
*g*_ m_	0.22 ± 0.05	**0.15 ± 0.03**	0.27 ± 0.03	**0.24 ± 0.02**
*V*_cmax_	213.75 ± 41.38	165.39 ± 16.80	199.54 ± 4.92	221.97 ± 15.86
LA:RA	0.74 ± 0.12	**0.53 ± 0.08**	0.63 ± 0.24	**0.28 ± 0.07**
LA:SA	8.72 ± 1.47	5.36 ± 0.77	6.56 ± 0.51^a^	3.35 ± 0.97^b^
Π_o_	-1.43 ± 0.37	-1.36 ± 0.39	-1.41 ± 0.18	-1.33 ± 0.10
TLP	-2.09 ± 0.29	-2.37 ± 0.45	-2.09 ± 0.12	-2.28 ± 0.19


*Numbers followed by different letters indicate significant differences between WW and WS plants for one genotype and bold numbers represent significant differences between the two genotypes for one irrigation treatment. Foliar N, leaf nitrogen (*g*N m^-*2*^); *g*_m_, mesophyll conductance (mol m^-*2*^ s^-*1*^); *V*_*cmax*_, maximum velocity of carboxylation (μmol m^-*2*^ s^-*1*^); LA:RA, leaf area divided by root area (m m^-*2*^); LA:SA, ratio between leaf area and sapwood area (cm^*2*^ mm^-*2*^); Π_*0*_, osmotic pressure at full turgor (-MPa); TLP, turgor loss point (-MPa).*

At whole-plant level, values of LA:SA and LA:RA ratios were always lower for GUA8 than GUA6 and for WS compared to WW. These differences were significant (*p* < 0.05) for the LA:RA ratio between the genotypes in WS plants, and between the irrigation treatments in the case of LA:SA for GUA8 (Table 3).

## Discussion

Our results suggest that whole-plant hydraulic allometry adjustments together with shorter-term leaf physiological responses allowed the GUA8 genotype to buffer the impact of the drought stress experienced, leading to a RGR that was less-affected by water stress compared to the other olive genotypes tested. At the whole-plant level, the observed fine tuning of the supply-demand hydraulic system made this genotype more capable of extracting and transporting water. Also, as the total leaf area was lower, water transport capacity on a leaf specific basis was higher. At the leaf level, the greater photosynthetic capacity in GUA8 WS than in GUA6 WS plants (higher *g*_m_ and *V*_cmax_ in WS plants, Table 3) also resulted in a slightly higher water-use efficiency for GUA8 under conditions of water stress (Figure 5C). Although it is difficult to estimate the below-ground biomass in adult trees grown under field conditions, more work is needed in adult trees to verify the patterns found in this study in juvenile olive seedlings growing in pots.

### Differential Response of Relative Growth Rate to Water Stress in Olive Genotypes

Our results showed that although *g*_s,max_ was only significantly reduced in summer, this decrease was sufficient to decrease RGR in different olive genotypes over a whole year (Figure 1). Such a long experimental period for this kind of study, coupled with long-term responses to soil and atmospheric drought as described here, are not usual. Although this experiment length adds value to the study, it could influence the results due to ontogenetic drift. As described by Rees et al. (2010), RGR is not totally size independent, because most plants become increasingly inefficient as they get larger because of self-shading, tissue aging, allocation to structural components, etc. However, such an effect is not likely to have happened in our study since there is no correspondence between the size of the plants (Table 2) and RGR (Figure 2) for either treatment. Despite belonging to the same species and sharing most of their water-stress response traits, differences were observed among the studied genotypes, with the GUA8 genotype having a significantly less-affected RGR as a result of decreased stomatal conductance in response to water stress (Figure 2). From the components of the RGR analysis, only physiological changes (NAR) were strongly and positively correlated to RGR_plant_ among the genotypes (Figure 3). Similar patterns have been found in other woody species (Galmés et al., 2005; Shipley, 2006), particularly under the high radiation of field experiments in comparison to laboratory or greenhouse experiments (Shipley, 2002). Other plant growth components did not show a common pattern of change among the genotypes analyzed, although in general denser roots and leaf tissues were found for GUA8 than for the other genotypes, which is consistent with GUA8 being less affected by water stress. The influence of the different components on the decrease in RGR imposed by drought conditions has been shown to be strongly dependent on the species in question, reflecting differences in response and adaptation to environmental constraints (Galmés et al., 2005).

### Coordinated Response of Hydraulic Properties and Leaf Gas Exchange to Water Stress

We further assessed relationships between, and differences in, physiological parameters that might influence gas exchange and thereby explain why the RGR of GUA8 was less affected by water stress than GUA6. At the leaf level, and for both genotypes, the net photosynthesis rate was shown to be mainly limited by stomatal conductance (Figure 4, 5) as demonstrated for many other species, given that stomatal closure is one of the earliest responses to drought and the dominant limitation to photosynthesis under mild to moderate drought conditions (Flexas and Medrano, 2002). The relationship between stomatal conductance and leaf hydraulic conductance was strong (Figure 4), thus adding to a growing body of evidence reporting the coordination between water supply and demand at the leaf level (Sack and Holbrook, 2006; Scoffoni et al., 2016). Leaf hydraulic conductance determines the efficiency of the coordination between water supply and demand, and hence, it may determine the degree that the stomata can remain open to allow photosynthesis. In that sense, leaf hydraulic conductance has been increasingly recognized to play a central role in determining plant performance and productivity (Brodribb, 2009; Flexas et al., 2013).

At the plant level, we observed changes in the hydraulic allometry (as proposed by Maseda and Fernández, 2006) of WS plants compared to WW plants, with morphological adjustment being more evident in GUA8. These changes involved a decrease of leaf area to sapwood and root areas, which may reflect a tuning of the hydraulic structure of these individuals to increase water extraction and transport capacity under conditions of water deficit, thereby improving the supply of water to the leaves and the leaf-specific hydraulic conductivity of the plant (Martínez-Vilalta et al., 2009; Martin-StPaul et al., 2017). This, in turn, helps to maintain stomatal conductance (Addington et al., 2006) and photosynthesis (Zhou et al., 2016). WS GUA8 showed a significant increase in root area to leaf area ratio compared to that seen in WS GUA6. This change could contribute to improved plant hydraulic efficiency by helping to maintain the plant water potential within a safe range, thereby reducing the risk of disruptive xylem embolism (Magnani et al., 2002) and a decline in below-ground hydraulic conductance (Johnson et al., 2018). In addition, olive plants have been shown to be very resistant to cavitation, including leaf xylem and coarse root xylem pathways (Rodriguez-Dominguez et al., 2018), so loss of xylem water transport capacity under our experimental framework were unlikely. However, pathways outside the xylem may have reduced *K*_leaf_ and, in turn, *g*_s_ (Scoffoni et al., 2017) under moderate water stress conditions.

Although homeostasis in response to a sudden perturbation can be achieved only through stomatal regulation, structural changes appear to play a central role in the plant’s adjustment to prevailing environmental conditions over periods of months to years (Magnani et al., 2002). Indeed, the LA:SA ratio was also highly correlated to stomatal conductance, although this was not the case for the LA:RA ratio. Despite the lack of association between LA:RA and *g*_s_, optimal allocation of resources between transpiring foliage and absorbing roots has been suggested to be coordinated with short-term regulation of *g*_s_ in response to drought (Magnani et al., 2002; Rodriguez-Dominguez and Brodribb, unpublished). A differential LA:RA response to water stress by the GUA6 and GUA8 genotypes, used in the path analysis, might underlie the lack of the relation between LA:RA and *g*_s_, as mentioned above. New advances in root hydraulics that are just beginning to emerge (Cuneo et al., 2016; Poyatos et al., 2018; Rodriguez-Dominguez et al., 2018) will bring new possibilities to explore the impact of changes in LA:RA on stomatal conductance.

### Carbon Balance at the Leaf Level

Despite *g*_s_ and *A*_N_ being very similar in the two genotypes, *g*_s_ was slightly higher in GUA6 than in GUA8, although this was not reflected in *A*_N_. This resulted in a better instantaneous water use efficiency for GUA8 than GUA6, which could be advantageous under conditions where water is scarce. Indeed, GUA8 exhibited leaf gas exchange traits which enhanced the net photosynthesis rate for a given *g*_s_. This was observed in terms of changes in *V*_cmax_ and *g*_m_. A larger *g*_m_ is an interesting solution for plants under water stress (Barbour et al., 2010; Flexas et al., 2016), since it reduces that drawdown in CO_2_ from the intercellular spaces to the chloroplastic sites of carboxylation, without an increase in transpiration. This is even more important if *V*_cmax_ has increased, as in the case of GUA8, since a higher *V*_cmax_ demands more CO_2_. Therefore, an orchestrated enhancement of both *V*_cmax_ and *g*_m_ is necessary to yield the desired goal of increasing *A*_N_ under water stress conditions. This physiological strategy has been reported as being typical of Mediterranean species with sclerophyllous leaves (Flexas et al., 2013; Peguero-Pina et al., 2015a, 2017). Moreover, the mechanism has not only been shown in angiosperms but also in gymnosperms (Peguero-Pina et al., 2015b), and is now accepted as a typical characteristic of species living in arid and semi-arid environments.

The high concentration of leaf N, as measured in this study for GUA8, confirms that this increase in nitrogen is not a mechanism for storing this macronutrient. The prime goal of the increase in N is directed to an enhancement of *V*_cmax_ and subsequently the *A*_N_. The increase of N is putatively driven by the decrease in SLA, since a larger mass is concentrated by leaf surface area. Foliar N is mainly allocated to the photosynthetic apparatus of the leaf (Rubisco, electron transport, and chloroplasts) (Evans, 1989). This obviously has a direct impact on *V*_cmax_ and *J*_max_, but it is also likely to affect *g*_m_. Although we have no data on the anatomy of the leaves, an increase of SLA and N have been reported to enhance the surface area of chloroplasts exposed to the intercellular spaces, thus improving the liquid component of mesophyll conductance, which is usually the most limiting factor for *g*_m_ (Tosens et al., 2012; Tomas et al., 2013; Flexas and Diaz-Espejo, 2015).

## Conclusion

We showed here that genotypes belonging to the olive species can exhibit different RGRs in response to water stress. Although differences among genotypes within species are usually smaller than differences among species, two main adjustments to improve the net photosynthesis rate were identified in one of the genotypes (GUA8) used in this study, allowing it to maintain or even increase growth rate under mild water stress conditions. First, at the whole-plant level, a hydraulic allometry adjustment took place as a result of the decrease in the ratios of the areas of leaf-root and leaf-sapwood, the latter being also strongly related to stomatal conductance. Secondly, at the leaf level we identified an increase in CO_2_ fixation for a given stomatal conductance that was brought about by an adjustment of traits optimizing CO_2_ fixation (higher mesophyll conductance and leaf N favoring maximum carboxylation rate). We also found that the leaf hydraulic conductance plays an important role in controlling stomatal conductance. Multi-scale studies such as the present one can be of great help to provide information on alternative opportunities to generate more drought-tolerant varieties.

## Author Contributions

VH-S, AD-E, and CR-D contributed to the conception and design of the study. VH-S organized the database, performed the statistical analysis, and wrote the first draft of the manuscript. PD-R, AD-E, and JC-F wrote sections of the manuscript. All authors contributed to manuscript revision, read and approved the submitted version.

## Conflict of Interest Statement

The authors declare that the research was conducted in the absence of any commercial or financial relationships that could be construed as a potential conflict of interest.

## Supplementary Material

FIGURE S1 Growth and water use traits of wild olive genotypes. Water consumption relative to leaf fresh weight (FW), total FW, shoot FW, root FW, and the root *vs.* shoot (R/S) biomass ratio was quantified in potted seedlings during the 2015 screening in selected genotypes from the subspecies *europaea* (ACZ8, ARC1, ACZ27, APR1, AJA18, AJA4, AJA1, ACO15, AJA6, ACZ4, ACZ9, ACZ1, ACO18, AMK34, ACZ10, ACO14, AMK5, AMK14, AMK16, ACZ5, AMK21, and AMK6), *guanchica* (GUA7, GUA6, GUA1, GUA4, GUA5, GUA8, GUA2 and GUA9), and *cuspidata* (CEH3, CEH9, CEH24, CEH6, CEH8). Bars are the average of six plants ±1 SE. Asterisks indicate significant differences between well-watered (WW) and water-stressed (WS) plants for each genotype (*p* < 0.05).
