# Supplementary figures and images for: Hydraulic Traits Emerge as Relevant Determinants of Growth Patterns in Wild Olive Genotypes Under Water Stress
Source: Front Plant Sci. 2019 Mar 13;10:291. doi: 10.3389/fpls.2019.00291 (PMC6424893; doi:10.3389/fpls.2019.00291)

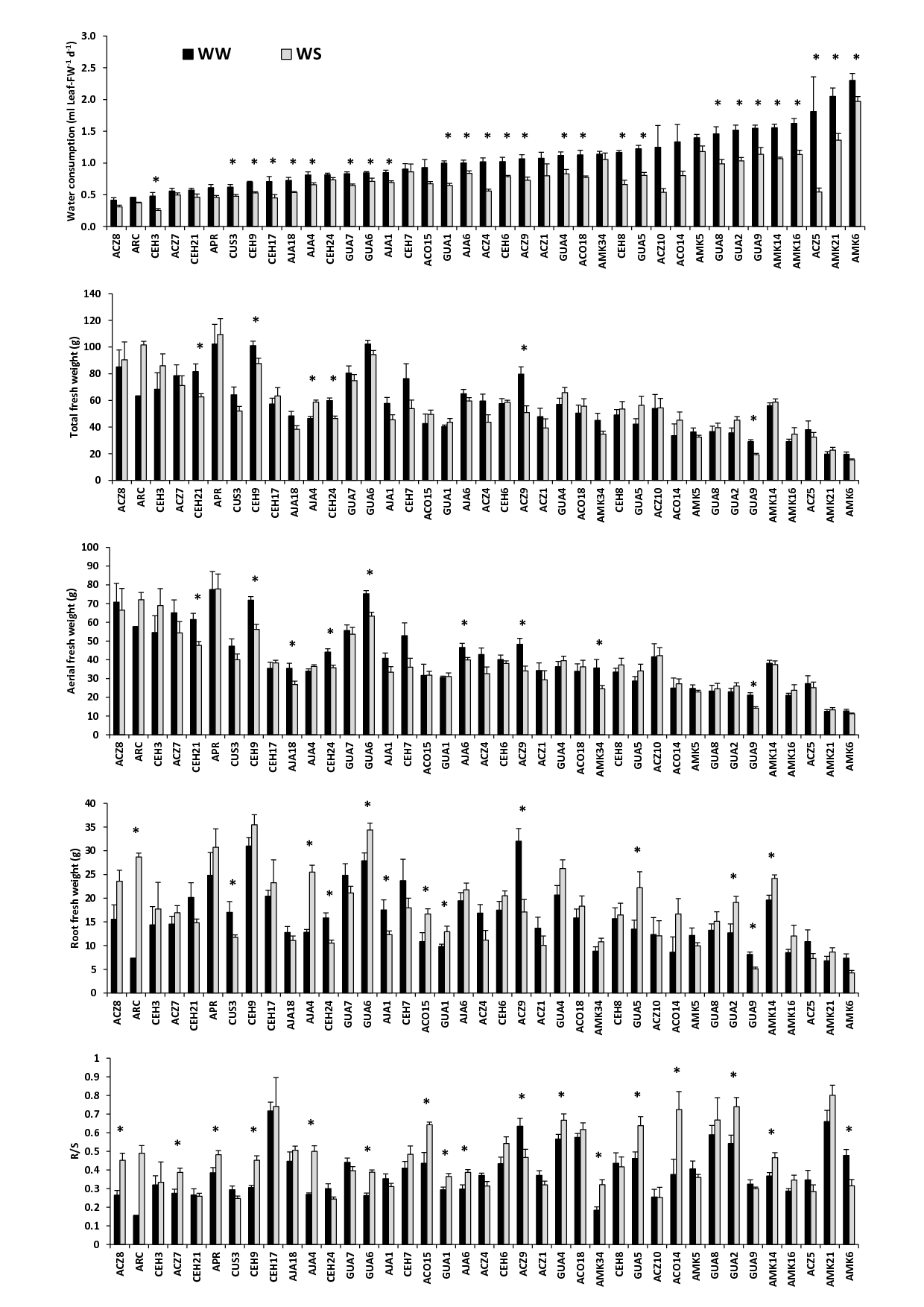

Supplement: FIGURE S1 — Growth and water use traits of wild olive genotypes. Water consumption relative to leaf fresh weight (FW), total FW, shoot FW, root FW, and the root vs. shoot (R/S) biomass ratio was quantified in potted seedlings during the 2015 screening in selected genotypes from the subspecies europaea (ACZ8, ARC1, ACZ27, APR1, AJA18, AJA4, AJA1, ACO15, AJA6, ACZ4, ACZ9, ACZ1, ACO18, AMK34, ACZ10, ACO14, AMK5, AMK14, AMK16, ACZ5, AMK21, and AMK6), guanchica (GUA7, GUA6, GUA1, GUA4, GUA5, GUA8, GUA2 and GUA9), and cuspidata (CEH3, CEH9, CEH24, CEH6, CEH8). Bars are the average of six plants ±1 SE. Asterisks indicate significant differences between well-watered (WW) and water-stressed (WS) plants for each genotype (p < 0.05). [file Image_1.tiff]
